# Book Reviews

**Published:** 1799-12

**Authors:** 


### CRITICAL RETROSPECT OF MEDICAL AND PHYSICAL LITERATURE.

### [ FOREIGN AND DOMESTIC. ]

### MEDICINE.

*Annales Instituti Medico-Clinici Wirceburgensis: Redegit & Observationibus Illustravit* J. N. Thomann, M. D. &c. vol. i. cum. v figuris aeri incisis. lxxviii & 229. pp. *Wirceburgi* apud A. M. Köl. 1799.

In this volume, which is written with perspicuity, the author communicates to us, 1st. A concise history of the origin and progress of that excellent institution, the magnificent hospital at Würzburg, founded by prince-bishop Julius, towards the conclusion of the sixteenth century, at first for the reception of two hundred patients. 2d. Meteorological observations. 3d. Lists of diseases from the 1st of April to the 31st of December, 1798; and, 4th. Medical observations on the most interesting cases.

As the last part will be chiefly interesting to the medical reader, we shall extract a few passages from the most remarkable cases, in the author's own words.

After giving a general description of the Intermittents which prevailed at Würzburg, in July last, and their treatment, the author makes the following remarks:—"Morbi in universum non multi fuere, maxima parte chronici hoc mense vulgares, quorum nonnullos, licet minus graves, notabilores tamen, hoc tempore grassantes exponam.

"Diarrhœa habitualis iure potentiis debilitantibus praegressis, praecipue purgationi nimiae adscribitur, quae saepissime a Medicis et Medicastris sine omni discrimine ordinatur. Non curant monita nostro tempore variis in libris novellisque literariis adlata, quibus abusus ac tumultuaria purgantium exhibitio damnatur; et, spernentes ulteriora rei medicae studia, sua methodo, cui iam ab ovo adsueti sunt, contenti, minime terrentur ancipiti experimento, etsi quotidie morbum purgantibus adaugeri, universi corporis organismi incitationem minui, morbosque, levi medicamine profligandos, in pertinacissimos ac gravissimos ruere videant.

"Sunt tamen prudentes Medici complures, qui doctrinam illam in variis Germaniae scholis depraedicatam, omnes fere morbos e sordibus primarum viarum derivandos esse, ad vanas et inanes phantasias referant, omni veritate et recta observatione destitutas. Si quae mala emeticum aut purgans exigunt, erunt forsan ab indigestione orta, neque febres, quas dicunt gastricas, ita adpellandas censeo, quarum quippe causa ac fons inter praecordia non latet, sed alibi potius quaerendus est, ut magis edocet verum organismi animalis studium, iudicium et experientia. Minime enim morbi illi antigastricis, neque solventibus, neque emeticis, neque purgantibus organismi animalis incitationem ac incitabilitatem devastantibus tolluntur.

Alius,

"Alius, de quo ſermonem faciam, morbus erat renum peculiaris adfectio a ſabuloſis et calculoſis concrementis ſuſcitata. Multifariis iam lithontripticis ſic dictis, uva urſi ab Haenio maxime, aqua calcis cum ſapone a Stephens, lixivio ſaponario a Iurin et Chittiks, alcali vegetabili ac minerali, aere fixo a Percival, Saunders, Hulme magnopere laudatis aliiſque remediis inſtitui experimenta: nullum vero votis magis reſpondit, quam aqua mephitica alcalina a Colborn et Falconer commendata. Abſit tamen, ut huic aliive remedio vires lithontripticas tribuam, i. e. virtutem, calculos in rene ſolvendi, aut deterendi, abradendi. Videtur potius grato ſuo ſtimulo urinae ſecretionem adaugere, qua poſtmodum ſabuloſa concrementa ac minores calculi una cum muco glutinoſo excernuntur. Aut forſan haec aqua alia et ſingulari gaudet, calculos depellendi, virtute ſub ſenſus non cadente, qualem multis adhuc medicaminibus tribuimus, quorum effectum, minime tamen effectus cauſam novimus. Quamquam ad expellendos calculos maiores ac perfecte formatos illud remedium nihil omnino conferre experti ſimus, plures tamen nobis innoteſcunt homines, calculoſis et ſabuloſis concrementis ac multis exinde ortis moleſtiis adfecti, aquae huius uſu reſtituti. Incommoda enim mox ceſſabant, atque per urinas multa alba glutinoſa materies ſedimento ſabuloſo per longum temporis intervallum cum quodam urethrae ardore eiiciebatur. Alba illa materies pauculo tempore ſibi relicta friabiles formavit rubros vel albidos lapillos, urinaque blandam ſenſim amplexa eſt indolem, neque in urinis amplius cernebatur arena: quibus perſtantibus ardor urethrae utique evanuit, et aegri poſtmodum eam recuperarunt ſanitatem, ut et poſt multos annos calculoſis adfectionibus immunes vixerint, et hodiedum vivant. Neque in morbo hoc calculoſo ſolum, ſed et in aliis renum, veſicae ac uretherum morbis, medicamen illud iuvare, immo mucum atque gluten vaſa renum excretoria obdurans illo reſerari, atque e corpore eliminari, veſicae ulcera, uretherum ac pelvis renum exulcerationes depurari ac ſanari obſervatum eſt. Quod ſi interea contingit, ut aut nimia mali intenſitate, aut partium deſtructione, aut labe quacunque alia corpus tenente ac depaſcente homo non liberaretur, immo periret, nihil id derogaret remedii praeſtantiae, quippe quum omnium vel optimorum remediorum ea ſit natura, ut intra certos tantum mali limites agant, et extra eoſdem agere nequeant. Sufficit etiam in eiusmodi caſibus eam remedii deprehendere efficaciam, ut, quum ſpes nulla adfulgere videatur, ſaltem inexſpectatum adferat levamen.

Aquae huius alcalinae iuxta formam Falconeri paratae aegrotans mane per tres vices uncias ſedecim ad viginti quatuor ſumſit. Quantitatem vero ſemper aegrotantis viribus ac ventriculi praeſertim robore metiebar. Ad quae diiudicanda niſi vitae ante actae ratio corporiſque conſtitutio ducerent, effectus aquae propinatae iuvabat, quam caute ſemper porrexi, ne digeſtio unquam laederetur, ventriculus gravaretur, anxietas, pectoris oppreſſio, cardialgia, colica, ventris inflatio, diarrhoea aliaeque orirentur moleſtiae, quas non raro ab immodico aquae uſu, nunquam a modico proveniſſe vidi. Eas tamen, ſi adparebant, uſus roborantium amarorum diſſipavit; nonnunquam ſtatim in initio ad praecavenda haecce incommoda cum aqua mephitica alcalina ſimul et roborantia exhibui." pp. 67—70.

Among the moſt inſtructive caſes are thoſe which the judicious author has illuſtrated with plates: viz. the hiſtory of an oſſification in the heart, heart, found after diffection; and a cafe of dropfy of the ovarium. Of thefe, we purpofe to give a detailed account to our readers, in a future Number.—At prefent, we fhall only take notice of a cafe of *emphyfema*, which is fufficiently important, both on account of the complicated variety of fymptoms, and the rational practice adopted by the phyficians of that hofpital. The particulars of this cafe are as follow:

" Piftor quidam, annos 44 natus et fanis ortus parentibus, corpore fat robufto laetaque a teneris fanitate ufus eft, nifi quod 12mo aetatis fuae anno per tres menfes vexaretur fcabie. Moleftum hoc exanthema adhibito unguento ex fulphure, Mercurio et oleo olivarum ita evanuit, ut ullas inde remanfiffe moleftias fibi haud meminerit. Ex coniugio fat infelici tres genuit proles cum uxore, quae ex cancrofo ulcere faciem occupante periit. Qua defuncta opibus exhauftus cum infantibus ipfi relictis vitam vivebat miferrimam, plerumque vagabundam. Quum 24to aetatis fuae anno in cella vinaria laborans atque multum refrigeratus magnam vini frigidi copiam avidis haufiffet faucibus, fubito moleftiam in pectore fenfit: horrorem fat vehementem infequebantur calor, languor, tuflicula ficca, fenfatio quaedam tendens ac premens in pectore, refpiratio difficilis et anxia. Increfcentibus noctu hifce fymptomatibus quaerit auxilium a Medico, et mox reftitutus fuit. Ex eo tempore, expofitus omnibus miferae ac vagae vivendi rationi adnexis calamitatibus, variis adfligebatur morbis, in quibus tamen adfectiones pectoris maximi femper fuerant momenti. Quum praeterea pro vitae fuae genere pondera levando pulmones adhuc magis exagitaret, accidit, ut in maiorem morbi traheretur opportunitatem. Quare omnio fere vernali et autumnali tempore a tufficula ab initio ficca, in pofterum autem fputis foluta, adfectum fe fuiffe refert. Quibus pectoris adfectionibus fe nonnunquam adfociavit cardialgia, quam ufu fpiritus vini vinive calidi profligare conabatur, quod remedium mox amavit tantopere, ut quotidie fere dimidiam vel etiam libram unam fpiritus vini biberet. Infelicem hanc confuetudinem firmavit magis Medicus quidam, qui aegro elixirium quoddam fpirituofum mane fumendum praefcripfit. Levatus inde paululum aeger lubenter huic confuetudini indulfit, minufque folicitus de fua valetudine aeftatis calore excalefactus aquam haufit frigidam, nec non data quavis occafione potibus fpirituofis ufus, hoc tempore tandem autumnali corripitur omnibus pectoris adfecti fymptomatibus fupra iam enarratis. Adpetitus praeterea fuit depravatus, atque fudoribus nocturnis fere diffluens aeger valde debilitabatur. Tuffis cum fputis e viridi flavis ac tenacibus mane e lecto furgentem valde anxium reddebat. Menfe Octobri ad fuam fuorumque fuftentandam vitam ruri circumvagus valdeque excalefactus, larga fubito pluvia per totum corpus madefactus, in proximum feftinans hofpitium calori fornacis proxime adfedit. Siccatus paululum aeris frigori fe denuo expofuit, iter in urbem profecuturus. Sed haud longe progreffus fubito collum quafi rigidum et tumens, totum caput graviter occupatum, inftantem quafi apoplexiam, pectoris oppreffionem et conftrictionem, et in latere thoracis dextro fenfum titillationis percepit. Viribus valde proftratis in urbem venit. Noctu fubitaneo totumque corpus percutiente frigore excitatur e fomno, quumque ob refpirationis difficultatem e lecto fefe adtollere cogeretur, capitis, trunci et extremitatum fuperiorum tumorem fentit ac gravedinem ingentem; palpebrae oculi dextri tumentes bulbum obtegunt. Infequitur calor corporis urens, fitis inexplebilis, orthopnoea. Altera die, 5ta Octobris, mifer mifer hic ad nofcomium confugit. Capitis et trunci corporis monftrofa confpicitur tumefcentia, facies rubedine fulgens eryfipelatofa, palpebrae utriufque oculi tumens ita, ut bulbi obtegerentur. Mammae earum mulieris inftar lactefcentis turgent. In capite, trunco et extremitatibus fuperioribus ftrepitus fub adtactu crepitans, ut in emphyfemate, fat bene diftinguitur; frigus praeterea et calor alternans, refpiratio difficilis, anxia, ftertorofa, fitum nonnifi erectum in lecto concedens aegro; tuffis frequens et ficca; fputa interdum fpumofa; pulfus parvus, debilis et frequens; cutis arida, fitis multa, alvus obftructa.

"Morbi caufam praeter tempeftatis mutationes ac refrigeria aeger nullam accufat: quare prognofin difcrimine plenam pronuntiantes praefcripfimus:

℞ Aq. flor. fambuc. Unc. feptem.
Vini antimon. Huxh. Dr. duas.
Spir. Minder.
Syrup. diacod. aa Unc. unam et dimid.
MDS. Omni hora cochlear unum.

"Adplicetur clyfma emolliens. Diaeta tenuis cum vino. Regimen calidum et ficcum.

"Ne ullum de aeris fub cute praefentia fuperfit dubium, fonticulum ad brachium dextrum pofuimus, quo levi preffione aer inter fanguinem eo loco adgregatum bullularum fub forma exiit.

"A 6ta ufque ad 9nam Octobris. Continuata hac medendi methodo eryfipelas faciei ut et febris evanefcit; fubfequente largo fudore, tumor capitis et reliqui corporis collabitur; ftrepitus tamen tactui fenfibilis atque pectoris adfectiones et tufficula cum fputis tenacibus e viridi flavis permanent: unde pulmonis dextri vomicam feu exulcerationes a neglectis praegreffis pectoris morbis et vitae genere fufpicati fumus. Ordinatur:

℞. Rad. polygal. feneg. Dr. duas.
coq. cum aq. font. libra una ad remanent.
Unc. feptem. Col. adde
Mell. depur. Unc. unam.
MDS. Omni bihorio cochlearia duo.

"Diaeta portio quadrans. Pro potu vinum aqua dilutum.

"Die 9na et 10ma. Omnia eadem, nocte fudor largus. Praefcribitur:

℞ Opii puri Gr. unum.
Rad. Ipecacuanh. Gr. dimidium.
Sacch. albi Dr. dimidiam.
M. f. pulv. div. in duas part. aequales.
DS. Mane et vefpere dofis una.

"Die 11ma et 12ma. Symptomata et ordinatio eadem.

"Die 13tia. Symptomata pectoris valde imminuta; fputa puriformia faciliime eiiciuntur; laffitudo inter largos per noctem fudores; emphyfema fere totum evanidum. Praefcribitur:

℞. Pulv. cort. peruv. Unc. unam.
Rad. polygal. feneg. Dr. duas.
coq. c. aq. font. libra una ad remanent.
Unc. octo. Col. adde
Mell. depur. Unc. unam.
MDS. Omni bihorio cochlearia duo.

Pro potu decoctum Lich. Ifland. Portio dimidia cum vino.

"A

" A die 14ta ad 18vam. Adfectiones pectoris valde imminutae; restat autem tussis cum sputis puriformibus. Hinc sublato emphysemate et faciei erysipelate aegrum declaravimus phthisicum, et post aliquod tempus viribus magis restauratis e nosocomio dimisimus."

---

*Notice of some Observations made at the Medical Pneumatic Institution: By* T. BEDDOES, M. D. 8vo. p. p. 47, price 1s. 6d. *Bristol* and *London.* Longman and Rees, 1799.

Dr. Beddoes commences this pamphlet with an explanation of the causes of delay, which have retarded the long expected reports from the Pneumatic Institution.

" At length, after some disgusts and much delay, we have it in our power to announce the first proceedings at the establishment, for applying chemistry to the elucidation of animal nature, principally by pursuing the connection between the properties of elastic fluids, and the conditions of life. By such an investigation, the public has been already too often told how much I consider it as practicable to advance *physiology*, the most interesting of the sciences, and *medicine*, the most useful of the arts. Intimately persuaded that immense improvements must, sooner or later, result from the inquiry, provided Nature be consistent with herself, and nothing doubting the truth of this, the fundamental postulate of all philosophy, I felt little discouraged by failures, which the presumption of sciolists has often busied itself in representing as decisive. For, who would suffer himself to be turned prematurely aside from an useful pursuit, by reptiles that plant themselves on the high road of improvement, and try to hiss back all who would advance? — Nor has the intelligent part of the public, I believe, been induced to regard as finished that which could not properly be said to be begun. For myself, among a multitude of reports, that prove nothing beyond the safety of the research, observing some far more favourable than could be expected from the excessive disproportion between the means hitherto employed, and the end in view, I incessantly persevered in urging the execution of the design.

" How widely this proceeding departed from that wary professional conduct, which, above all things, avoids committing itself by any measure of striking singularity, and is content with the eternal repetition of processes, from which nothing of advantage is expected, and nothing gained, I could not but be conscious. The present was, perhaps, the first example, since the origin of civil society, of an extensive scheme of pure scientific medical investigation.

" To have engaged in it, however, either without a sufficient fund, or the most able assistance, would have been to do a good cause the most lasting of injuries, supposing (what I have long sincerely believed) that extensive benefit may result from the undertaking. The qualifications of a superintendant, were, indeed, of still greater importance than the amount of the subscription. In some hands, the largest sum would have been utterly unproductive. And the acquisition of a properly qualified associate, might be considered as more than virtually doubling the fund; since it is the prerogative of superior talents, to accomplish great purposes by small means."

Dr. BEDDOES gives an account of the effects produced by breathing a certain modification of the *dephlogisticated nitrous gas* of Dr. PRIEST-

LEY, ley, which are aftonifhing enough, and will, doubtlefs, produce no common fenfation in the minds of phyfiologifts.

The prefent publication, however, is only a notice of a regular quarterly account of the Inftitution, and its future profpects. This periodical work the author here announces, under the title of *Refearches concerning Nature and Man.*

